# Does Green Space Really Matter for Residents' Obesity? A New Perspective From Baidu Street View

**DOI:** 10.3389/fpubh.2020.00332

**Published:** 2020-08-07

**Authors:** Yang Xiao, Yuhang Zhang, Yangyang Sun, Peihong Tao, Xiaoming Kuang

**Affiliations:** ^1^College of Architecture and Urban Planning, Tongji University, Shanghai, China; ^2^Shanghai Tongji Urban Planning and Design Institute, Shanghai, China

**Keywords:** green space, eye-level, street view image, BMI, Shanghai

## Abstract

Despite a growing literature on the topic, the association between neighborhood greenness and body weight is inconsistent. The objective of this research is to examine the association between neighborhood greenness and residents' obesity levels in a high population density area. We accounted for three greenness features: green access, green exposure, and view-based green index. We used the novel technique of deep convolutional neural network architecture to extract eye-level information from Baidu Street View images to capture the urban vertical greenness level. The research involved a survey with 9,524 respondents from 40 communities in Shanghai. Generally, we found all aspects of horizontal greenery, vertical greenery, and proximity of green levels to be impactful on body weight; however, only the view-based green index consistently had an adverse effect on weight and obesity.

## Introduction

Scholars have found links between the built environment and health-related outcomes, including mental health (1), cardiovascular outcomes (2), and mortality (3) through a socio-ecological approach, emphasizing individual interactions with the built environment (4, 5). There has been an increasing focus on the relationship between green space and residents' body mass index (BMI), as being overweight or obese poses many health risks, including diabetes, heart disease, and certain forms of cancer. Indeed, obesity has become a global public health issue. As reported, the number of obese men and women rose from 232 to 304 million from 1975 to 2014 (6). Greenness can promote people's physical activity, which can facilitate healthy weight management (7). Thus, in general, good neighborhood greenness levels have been associated with a lower likelihood of being overweight or obese. However, the association between greenness and body weight seems less consistent than believed (2, 8–10). For instance, Cummins and Fagg (2012) found that green space was associated with increased chances of being overweight and obese, but this relationship has only been found in urban areas in 2000–2003 (11). There is no significant correlation between green space and BMI (12, 13), and the health effects of urban green spaces may vary according to gender, socioeconomic profile, and local context (14, 15).

With increasing evidence of an association between neighborhood greenness and resident weight status, some studies examining the association between eye-level neighborhood greenness and people's BMI have emerged (16, 17). The present measurements of the health effect of green space mostly rely on green exposure and access; a potential reason for such inconsistent findings is the differing approaches used to measure green space features (8). Specifically, most epidemiologic studies have employed satellite-based vegetation indices (normalized difference vegetation index, NDVI) or land-use databases (18), whereas urban geographers have preferred the measurements of proximity (e.g., nearest distance) and gravity (19, 20). With the emergence of ICT, individual-level and street-level data from sources such as Google Street View images may provide new opportunities to move beyond 2D-based greenness measurements (21, 22). In this context, the primary aim of this research is to explore the associations of eye-level greenness and residents' obesity in China, since China is facing rising rates of overweightness and obesity among its citizens (23). China's obese population exceeds that of the United States and ranks first in the world (6). We used Shanghai as the study area since it has a high population density and a green space disparity issue (24). This research was based on a survey of 9,524 respondents from 40 communities in the metropolitan area. We used the technique of deep convolutional neural network architecture to extract eye-level information from Street View images to calculate the visible green index at a vertical level. Additionally, we also considered the normalized difference vegetation index (NDVI) and the accessibility of urban parks to assess both the size and service effects of green space. We are particularly concerned about the relationship between different types of greening levels and obesity.

## Literatures Review

### Built Environment and Public Health

For quite some time, people did not pay attention to the impact of the built environment on residents' health, focusing more on the role of traditional health services. They did not begin to expand the scope to consider the factors affecting residents' health until the 1970's. Whitehead and Dahlgren claimed that the key factors affecting health include genetics, lifestyle, social economy, and environment (25). Evans believes that the built environment can directly affect mental health through six aspects: housing conditions, facility layout, living density, sound environment, air quality and lighting, and regulation and self-control. Mental state, affected by social support and healing, indirectly affects mental health (26). Considering the variety of influencing factors and the complexity of the influencing mechanism, the main influencing factors on resident health in the built environment can be summarized as follows: social environment, physical environment, and access to health and social services (27).

The green space can be defined as follows. Natural vegetation and artificial vegetation are the main forms of land use in urban areas. Green space comprises two types of land: land used for greening within the scope of urban construction land and the area outside the urban construction land that has a good green environment, which positively affects ecology, landscape, and residents' lives. As one of the most critical components of urban space, urban green space can reflect the quality of a city's life to a certain extent, and it is of great significance to the sustainable development of urban ecological environment and the support and promotion of residents' health and well-being. Urban green space provides a variety of ecosystem services (28). Jiang explained the influence mechanism of urban green space on public health in terms of five aspects: promoting physical exercise, soothing mental stress, reducing mental fatigue, providing ecological products and services, and enhancing social capital (29). It is also believed that green space promotes health by reducing stress and remodeling cognition, enhancing physical activity, promoting social interaction, reducing noise, regulating temperature and humidity, and filtering air pollution (10). Other researchers have further concluded that the main health effects of green space are reduced harm to the physical environment, reduced physiological and psychological stress, and promoted health-related activities (such as exercise and social interaction) (30).

With increasing evidence of the association of neighborhood greenness and resident's weight status, there are also some studies examining the association between eye-level neighborhood greenness and people's rising BMI (16, 17). Furthermore, a study using GSV data of Hong Kong shows that children's BMI is lower where the greenery is better around the school environment (16). Li and Ghosh (2018) from Cleveland, Ohio, USA found that street greenery has a stronger effect on women's BMI than that of men. Practically, many green-health-related studies use street view images (17). Lu (2019) used GSV data to determine the effect of eye-level street greenery on physical exercise (31). Helbich et al. found that the depression of the elderly in Beijing can be prevented with a better view green index (32). By comparing GSV data with green space data of other scales, GSV data reflect another special characteristic of green space and provide a new perspective for the study of the relationship between green space and public health (33).

### The Association of Greenness and Obesity

Obesity has been a widespread concern shared by many scholars because of the high risk it poses toward causing various serious chronic diseases. Moreover, there have been studies on the relationship between green space and obesity. Some studies have shown that the spatial elements of urban green space relating to obesity include accessibility, scale, frequency of use, and vegetation cover. Sarkar believes that the accessibility of green space is significantly negatively related to BMI, which may be due to the improvement of utilization efficiency of green space with high accessibility and the further reduction of the BMI level by residents using it for physical exercise (34). A study from Denmark (7) shows that access to a garden or living a short distance from green areas is associated with a lower likelihood of obesity. Halonen et al. conducted an 8-year study and found that living far from usable green areas or waterfront in urban areas increases the risk of overweightness (35). Mowafi et al. (2012) found that when the socio-economic status changed, there was no significant correlation between the accessibility of green space and BMI, and the main cause of obesity may be the deficiency of a diet structure of low-income groups (12). Cummins and Fagg (2012) analyzed the data from 2000 to 2007 in the UK and found that the green space scale was significantly related to BMI (11). A study using Dutch national health survey subjects (36) showed that NDVI surrounding greenness within a 300-m buffer zone in residential areas was significantly negatively correlated with overweightness. Tilt et al. considered NDVI and accessibility together and found that in areas with better vegetation cover, the objective accessibility of green space was negatively correlated with the BMI of residents (37). Relationships among green space attributes and BMI varied with age and gender. Green space composition and contiguity were related to BMI for some groups (15).

### Greenness Metrics

The mechanism of green space's influence on health is still controversial in the existing research. First, some active leisure activities that may have positive effects on health sometimes do not occur in the green space. For example, in high-density cities such as Hong Kong, active leisure activities rarely occur in green space because leisure space requires more hard-paved surfaces (38). Further, Fong et al. indicate that there may be some deviation in the analysis results using NDVI as the basis for measuring the spatial level of urban green space, owing to the lack of information on natural space quality and the detailed information of tree species or other vegetation (30). Land use data, used to measure the characteristics of green space, sometimes have their defects; they may not provide small-scale vegetation information, including small gardens. Finally, because of the different land classification methods used in different regions, the consistency and comparability of the unified global measurement method of NDVI are lacking, so it is difficult to realize the use of land use data in cross-regional research (10).

Research based on the health benefits of urban green space is well-established but is still in the early stage of development and progress (38). The evaluation system of urban green space spatial elements and the methods of obtaining and processing spatial data are also developing. Feng et al. (2010) claimed that there are differences among the results of a large number of empirical studies, which can be attributed to different methods of measuring green space (8). The spatial data of urban green space are generally sourced from government databases. The accessibility, scale, and density index of green space can be obtained by spatial analysis and processing urban traffic network and land use data. The frequency of green space and the diversity index of spatial utilization can be obtained by field investigation, observation, interview, and GPS positioning sensing. Through the analysis and processing of satellite remote sensing images, the green space index composed of vegetation spectrum, such as NDVI, can be obtained. With the development of 3 s technology, namely remote sensing, geographic information system (GIS), and global positioning system (GPS), the application of space technology, sensor technology, satellite positioning, navigation technology, and digital technology in the collection, processing, and analysis of spatial data of urban green space has been realized. Ye et al. (2018) collected and extracted Google Street View images through a machine-learning algorithm to obtain the View Green Index (VGI) to realize the accurate measurement of visual greening (39).

In summary, although there have been many studies on green space and residents' health, the existing studies cannot effectively reveal the impact of green space on urban health. There are many evaluation systems for green space and health status themselves, and the results obtained by applying different evaluation criteria in different research areas vary. Additionally, there are a few existing research studies that examined the health of green space based on the VGI. Most studies used the NDVI and other indices alone; when using the indicators to reflect the spatial distribution of green space, it is difficult to depict the whole picture of green space characteristics, as street scene greening is more closely related to the daily life of residents. Therefore, using the VGI to determine residents' BMIs as an example, this study examines the influence of green space on urban health. In this study, it was assumed that the green space represented by the VGI has an influence on residents' BMIs, which is different from that measured by the NDVI and spatial distance. The sensitivity varies across populations.

## Study Area and Method

### Data Source

**Figure 1:**
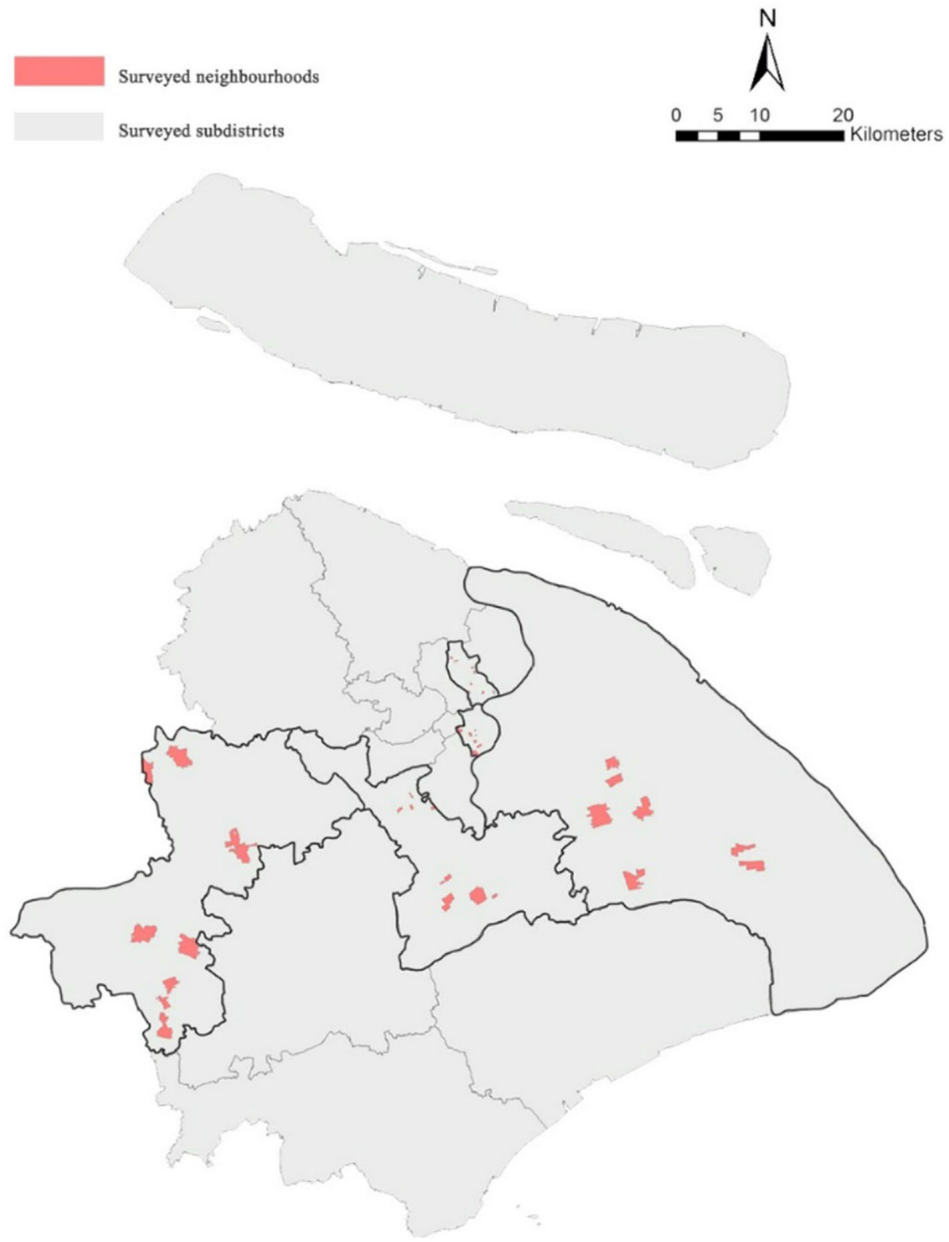
Location of research space unit in Shanghai.

Data on urban residents come from the World Health Organization's Global Aging and Adult Health Data (Sage, wave 1), which were obtained from 40 communities and 9,524 samples in Shanghai. The communities' areas in our study ranged between 0.022 and 5.607 km^2^, and their average size is 1.453 km^2^. These samples come from Huangpu District, Hongkou District, Minhang District, Pudong New District, and Qingpu District of Shanghai. Based on the principle of sample heterogeneity, these areas were randomly selected, and 40 communities were divided according to the size and type of communities in different towns or streets. After the screening of effective data, 40 communities and 8,988 samples were designed. The survey collected community information and personal information of residents. Community information included community location, whether that be an urban or another type of community; personal information of residents included information such as age, gender, income, marital status, profession, education level, and so on. The geographical distribution of the community is shown in Figure 1. In this study, the height (m) and weight (kg) of residents were calculated to reflect the level of obesity. The higher the indicator of the BMI, the more likely the residents are to be obese, and the unhealthier they are. The formula for calculating the BMI is as follows:3

(1) $$\text{BMI}=\frac{\text{Weight}}{\text{Heigh}{\text{t}}^{2}}$$

In 2003, the Ministry of Health of China introduced a new method to classify the BMI as per the specific traits of the Chinese population: 18.5 < BMI ≤ 23.9 indicates normal weight, 24 ≤ BMI indicates overweight, 24 < BMI ≤ 27.9 indicates pre-obesity, and 28 ≤ BMI indicates obesity. Based on this, we divided the BMI of residents into three grades: normal (18.5 < BMI ≤ 23.9), overweight (23.9 < BMI ≤ 27.9), and obese (28 ≤ BMI).

### Neighborhood Greenness Measurement

#### View Green Index

**Figure 2:**
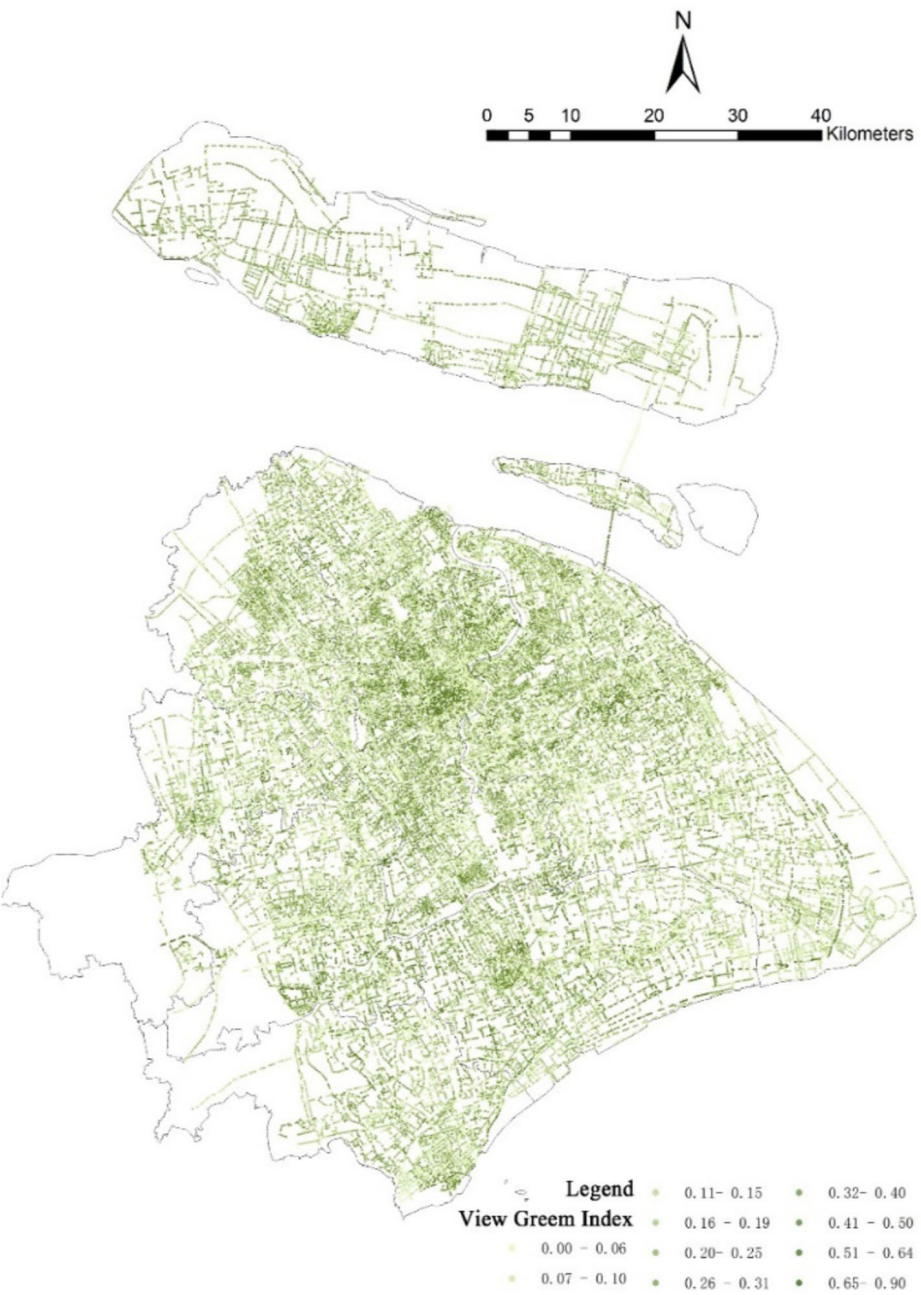
The distribution of View Green Index collected from street image in Shanghai.

In this study, we used the method of Ye et al. (2018) to calculate the VGI; urban roads were selected based on ArcGIS 10.3, and the 50-m interval was used to select points on the road. After obtaining the coordinates of the selected points, we used the Baidu map API to grab the street view image of the sampling points, thus obtaining complete street view data. We then imported these data into the open-source project SegNet, used the image segmentation methods based on super-pixels to interpret the image and classify it into color categories through the decoder, and finally analyzed the VGI data of Shanghai according to the calculated color proportion results. The ratio of green plant pixels to total pixels in four selection point images was used to evaluate the street greening level of the point (39), as follows:

(2) $$\text{VG}{\text{I}}_{\text{i}}=\frac{\sum_{\text{j}=1}^{4}\text{GreeneryPixel}{\text{s}}_{\text{ij}}}{\sum_{\text{j}=1}^{4}\text{TotalPixel}{\text{s}}_{\text{ij}}},$$

where *i* is the selected street view point; *j* refers to separate graphs; *GreeneryPixels* is the number of green pixels in point *i* graph *j*; and *TotalPixels* is the total number of pixels in point *i* graph *j*. In this study, a 1,000-m buffer was drawn based on the community center. The mean values of the VGI of all selected street view points in the buffer range were calculated to evaluate the greening level of each community using the VGI, as shown in Figure 2. The research radius of 1,000 m was determined by consulting many previous works of literature (31, 40, 41). Because walking is how residents come into contact with green space and the average daily walking time of most residents is 10 min, within 1,000 m, people will choose walking first (42). We also considered that only 6 out of 40 communities have an area of more than 3.142 km^2^; the buffer zone with a radius of 1 km can cover most of the community.

#### Greenness Exposure Index

**Figure 3:**
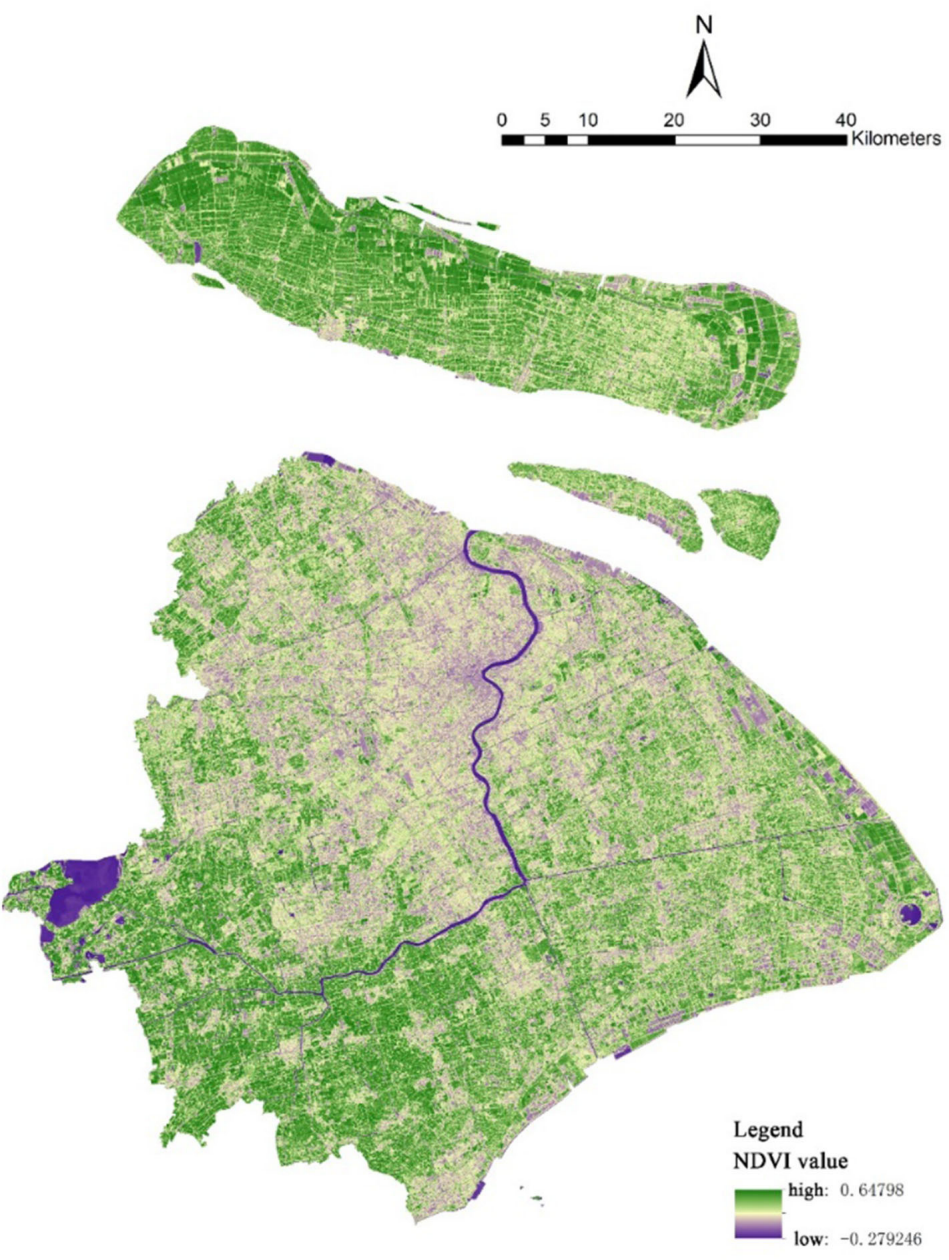
Remote sensing image of NDVI value distribution in Shanghai.

The NDVI is calculated from the visible and near-infrared light reflected by vegetation. Healthy vegetation absorbs most of the visible light that hits it and reflects a large portion of the near-infrared light. Unhealthy or sparse vegetation reflects more visible light and less near-infrared light. The relationship is expressed mathematically as follows: NIR is the near-infrared band, RED is the red band; the NDVI value range is (-1, 1). The larger the value, the higher the vegetation cover level. Weier and Herring (2000) classified the NDVI into the following grades:−1–0 represents a water body, 0–0.1 represents rock, sand, and snow plain (barren), 0.2–0.3 represents bush and grassland, and 0.6–0.8 represents temperate and tropical rain forests (43). The NDVI data belong to the band 4 RED and band 5 NIR, as recorded by the operational land imager of Landsat 8 satellite in Shanghai. The resolution is 30 × 30 m. Using the ENVI5.1 platform, radiometric calibration, atmospheric correction, and other means of pre-processing the original data and the NDVI normalization processing calculation tool, the NDVI data of Shanghai and its distribution remote sensing image were obtained (Figure 3). Like the VGI, we calculated the average NDVI in a 1,000-m buffer of communities to evaluate the NDVI status of communities representing greening.

(3) $$\begin{array}{l} NDVI=\frac{NIR-RED}{NIR+RED} \end{array}$$

#### Park Accessibility

**Figure 4:**
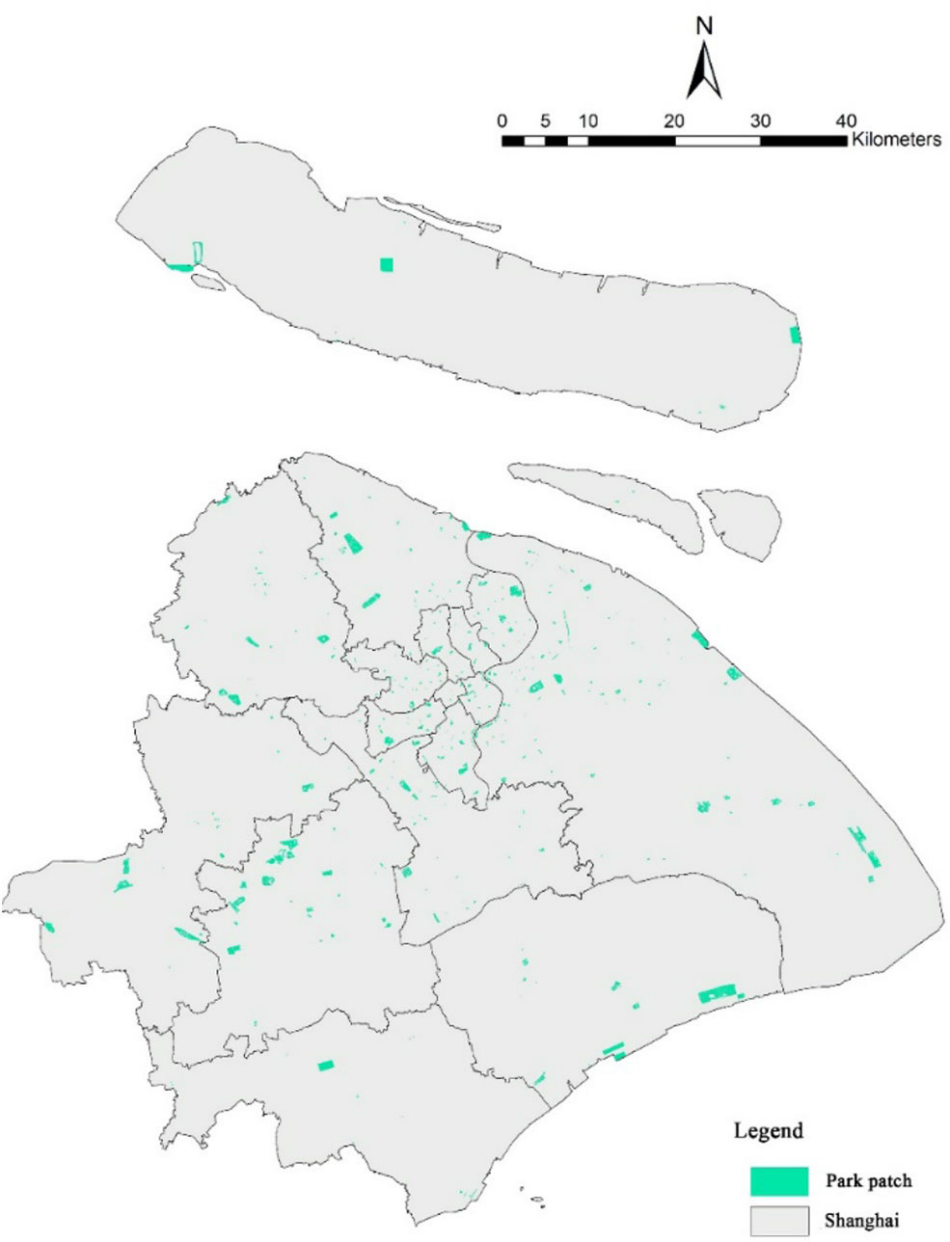
Geographical distribution of urban parks in Shanghai.

City parks can provide a suitable environment for leisure and sports activities. Parks are usually used as substitutes for open green spaces in research (39). A community's distance from the city park can represent the accessibility of the park for the community and reflect the contribution of park green space to the health of residents. Using ArcGIS, the nearest distance analysis of existing green space and community boundary was performed, and the nearest distance from the community to the park boundary was obtained. Meanwhile, we calculated the total area of the park in the 1,000-m buffer zone to obtain the scale of green space in the residential environment. The urban parks' data of Shanghai come from its cadastral map, which includes geographic information of 416 urban park patches in Shanghai (Figure 4).

### Model Specification

Our main purpose was to explore the influence of green space on overweightness or obesity. The “normal” BMI class was used as a reference group, and the other two groups were “overweight” and “obesity.” Since our dependent variable is a multinomial and ordinal category variable based on the consideration of green space in different communities, there are no fixed efforts on health. Therefore, the statistical model used in this study was a two-level multilevel mixed-effects ordered logistic regression. The model can be written as follows: where for a series of *M* independent clusters (residential district), and conditional on a set of fixed effects *X*_*ij*_, a set of cutpoints κ and a set of random effects *u*_*j*_, *z*_*ij*_ are the covariates corresponding to the random effects.

(4) $$\text{Pr}({\text{y}}_{\text{ij}}=\text{k}|\kappa ,{\text{u}}_{\text{j}})=\text{H}(\kappa_{\text{k}}-\beta {\text{X}}_{\text{ij}}-{\text{z}}_{\text{ij}}{\text{u}}_{\text{j}})-\text{H}(\kappa_{\text{k}-1}-\beta {\text{X}}_{\text{ij}}-{\text{z}}_{\text{ij}}{\text{u}}_{\text{j}})$$

where *j* =1,…, *M* clusters (in this case, 20), with cluster *j* consisting of *i* = 1,…, *n*_*j*_ observations (in this case, 8,988). The cutpoints κ are labeled κ_1_, κ_2_,…,κ_−1_, where K is the number of possible outcomes (in this case, 3). *H(…)* is the logistic cumulative distribution function that represents cumulative probability. Controlled variables reflect the community and personal characteristics of residents, including age, gender, marital, education, income, job, and location of communities. We also added the variable of self-rated health, which is composed of the Likert 5 scale. The results include the subjective scores of residents on their overall health: 1 “very unhealthy,” 2 “unhealthy,” 3 “general,” 4 “healthy,” and 5 “very healthy.” In this study, “Age” indicates whether the residents are over 60 years old, according to the WHO age classification standard, where 1 refers to “beyond 60” and 0 refers to “under 60.” “Gender” is the dummy variable of residential gender, where 1 refers to “male” and 0 refers to “female”; “Marital” is the dummy variable of resident marital status, where 1 refers to “married” and 0 refers to “otherwise”; “Education” is the dummy variable of residential education, where 1 refers to “middle school and above” and 0 refers to “otherwise”; “Income” is divided by whether the income of residents accounts for the top 25% of the total sample, where 1 refers to “yes” and 0 refers to “no”; “Job” is the dummy variable of employment, where 1 refers to “employed” and 0 refers to “otherwise”; “Place” is the dummy variable of communities, where 1 refers to “urban area” and 0 refers to “rural area.”

## Empirical Results

### Descriptive Results

In Table 1, we found that people with a normal BMI accounted for nearly half of the sample and that people who were overweight were twice as common as those who were obese. In terms of age, 50% of the samples were over 60 years old, meaning that the majority of the elderly population were included in the sample. The distribution of gender was relatively even, with females being slightly higher in number. The majority of the samples had spouses. In terms of individual socioeconomic characteristics, the education level of most people was lower than that of junior high school, which is understandable in combination with the fact that most were elderly. It was found that the economic conditions of residents were not too poor, which may be because nearly half of the sample members were still employed while others possibly had other sources of income. It was gratifying that most people were satisfied with their overall health, but 1 in 10 people still perceived their health as not ideal.

**Table 1:** Descriptive statistics of variables.

	***N^a^***	**%*^b^***
**Dependent variable**		
BMI Class (Mean = 0.665)	8,988	100.00
Normal	4,228	47.71
Overweight	3,493	28.86
Obesity	1,267	14.10
**Sociodemographic variables**		
Age (Mean = 0.501)	8,988	100.00
Under 60	4,490	49.96
Beyond 60	4,498	50.04
Gender (Mean = 0.459)	8,988	100.00
Female	4,861	54.08
Male	4,127	45.92
Marital (Mean = 0.848)	8,988	100.00
Married and cohabitation	7,621	84.79
Otherwise	1,367	15.21
**Individual SES variables**		
Education (Mean = 0.273)	8,988	100.00
Middle school and above	2,453	27.29
Otherwise	6,535	72.71
Income (Mean = 0.312)	8,988	100.00
Top 25%	2,801	31.16
Otherwise	6,187	68.84
Job (Mean = 0.448)	8,988	100.00
Respondent was employed	4,035	44.89
Otherwise	4,953	55.11
Place (Mean = 0.565)	8,988	100.00
Urban	5,087	43.40
Rural	3,901	56.60
**Health**		
Self-rated health (Mean = 3.515)	8,966	100.00
Very unhealthy	69	0.67
Unhealthy	751	8.37
General	3,368	37.56
Healthy	4,080	45.51
Very healthy	707	7.89
**Green Space**	**Mean**	**Std. Dev**.
NDVI (obs = 8,988)	0.213	0.076
VGI (obs = 8,276)	0.208	0.086
Area of Parks (obs = 8,988)	37,780.49	72,072.11
Nearest distance to parks (obs = 8,988)	1,813.429	2,163.851

a *N represents actual number of observations*.

b *Percentages reflect sampling weights from complex survey design*.

Regarding green space, the average NDVI of the research communities is low, which indicates that the vegetation coverage is poor. NDVI remote sensing image (Figure 3) can also directly reflect this point; that is, only the communities in Qingpu District have a higher vegetation coverage while other communities have a lower vegetation coverage due to a higher urbanization level of the built environment. Considering that some studies show that 15% of the VGI is the minimum acceptable value and that 25% of the VGI offers the most comfortable appearance, we can claim that the average VGI of the research communities is in the middle level (44). Overall, the research buffer zones cover a large area of parks, but the average nearest distance between the community center and the park is 2 km, which is a relatively long distance.

Our research hypothesis is that the impact of the green vision rate on residents' health is different from that of green space from other perspectives, and the impact is also different in different populations. So, we first build a basic model to analyze the health differences of different people and then add different green features from different perspectives to see whether green space impacts residents' health and whether it has a different impact on different people. Based on equation 4, first, we introduced the control variables to form the basic model (Model 0). Considering that the NDVI, the park area, and the nearest distance from the community to the park also reflected the state of green space around the community, they are described as per the characteristics of land cover, which is different from the evaluation of green space from the vertical direction by the VGI. Therefore, it is necessary to compare the effect of the VGI and the other three green space evaluation indexes on the BMI.

In the basic model, two variables, “distance to the park” and “park area within 1,000-m buffer zone,” were added to analyze the impact of park conditions and living environment on the BMI of residents through model (1); adding the variable “NDVI of 1,000 m buffer zone,” we obtained model (2) to analyze the living environment vegetation cover's effects on the BMI. “VGI of 1,000-m buffer zone” was used as an independent variable to construct model (3) to observe the influence of green space from the three-dimensional perspective represented by the VGI on the resident BMI. Finally, we added all the variables representing the green space level and the interaction terms of income level with park distance and the NDVI to analyze whether the effect of park conditions and vegetation coverage level on the BMI is affected by income (Model 4), because the residents' choice of community is usually independent of the VGI but in consideration of community vegetation coverage and surrounding urban parks.

### Regression Results

The results of the multilevel mixed-effects ordered logistic regression are shown in Table 2. As depicted in Model 0, the male and the married individuals tended to become overweight/obese, which may be due to the impact that emotional state has on people's lifestyles. The more educated the people are, the less likely they are to be overweight/obese. This is because people with good education may have better eating habits. People without work are more likely to be obese than those with work. People without work may have a lot less physically intensive activity. People with better self-rated health are more likely to be of normal weight, which may be due to how the obesity constitution has brought some negative health effects on people who have worse self-rated health.

**Table 2:** Results of multilevel mixed-effects ordered logistic regression model.

	**Model (0)**		**Model (1)**		**Model (2)**		**Model (3)**		**Model (4)**	
**Independent variable**	**Dependent variable**		**Dependent variable**		**Dependent variable**		**Dependent variable**		**Dependent variable**	
	**BMI (3 classes)**		**BMI (3 classes)**		**BMI (3 classes)**		**BMI (3 classes)**		**BMI (3 classes)**	
	**Coef**.	**Std. Err**.	**Coef**.	**Std. Err**.	**Coef**.	**Std. Err**.	**Coef**.	**Std. Err**.	**Coef**.	**Std. Err**.
Age	0.035	0.044	0.038	0.044	0.039	0.044	0.069	0.046	0.082	0.048
Gender	0.123^**^	0.041	0.122^**^	0.041	0.122^**^	0.041	0.142^***^	0.043	0.138^**^	0.045
Marital	0.168^**^	0.058	0.172^**^	0.058	0.172^**^	0.058	0.150^**^	0.061	0.108	0.063
Education	−0.243^***^	0.051	−0.247^***^	0.051	−0.247^***^	0.051	−0.237^***^	0.051	−0.253^***^	0.053
Job	−0.111^*^	0.047	−0.105^**^	0.047	−0.100^**^	0.047	−0.112^**^	0.049	−0.115^**^	0.052
Place	0.052	0.080	−0.001	0.090	−0.052	0.097	0.099	0.086	0.110	0.098
Income	−0.042	0.046	−0.043	0.046	−0.045	0.046	−0.053	0.047	−0.034	0.061
Self-rated health	−0.076^**^	0.027	−0.076^**^	0.027	−0.076^**^	0.027	−0.081^**^	0.028	−0.102^***^	0.030
Distance			−0.004^**^	0.002					−0.005^***^	0.014
Area 1,000 m			0.067	0.043					−0.004	0.004
NDVI 1,000 m					−1.038	0.558			4.784^**^	2.156
VGI 1,000 m							−1.019^**^	0.406	−1.298^***^	0.404
income_distance									0.005^***^	0.001
income_ndvi									−0.354	0.202
	Wald chi^2^ (8) = 63.49		Wald chi^2^ (10) = 70.30		Wald chi^2^ (9) = 66.89		Wald chi^2^ (9) = 74.41		Wald chi^2^ (14) = 96.78	
	Prob>chi^2^ = 0.0000		Prob>chi^2^ = 0.0000		Prob>chi^2^ = 0.0000		Prob>chi^2^ = 0.0000		Prob>chi^2^ = 0.0000	
	*N* = 8,966		*N* = 8,966		*N* = 8,966		*N* = 8,257		*N* = 7,583	

*** *p < 0.001*,

** *p < 0.01*,

* *p < 0.05*.

Models 1–3 estimated the impact of green space from different angles on residential health. The coefficient of “distance” was tested to be negative and significant at the 1% level, while the coefficient of “park area” was not significant in Model 1. It was found that the longer distance from parks relieves the risk of obesity or overweightness. This result seems to contradict our existing cognition of green space. Considering the large number of elderly people in the study, the reason for this result may be that the elderly generally do not use the park for physical exercise, but tend to rest and engage in social activities, so the walking process to and from the park has an impact on their physical health (34). In Model 2, the coefficient of the “NDVI” variable was not significant; that is to say, no direct correlation between the NDVI and residents' health was found, which is inconsistent with our expected results, because we initially thought that individuals surrounded by higher NDVI spots were more likely to not be overweight. In Model 3, the “VGI” yields negative and significant coefficients at the 10% level, indicating that growth in the VGI helps obesity control. The addition of variables of green space hardly changed the influence of other control variables. It is noteworthy that when comparing the basic model results, despite significant coefficients of green space-related variables, the coefficient symbol of “place” has changed in the model with urban park and vegetation coverage as independent variables, indicating a latent moderation effect in variables. But the coefficient symbol of “place” has not changed in the model with the VGI as the independent variable. We thought that the variable “income” that affected residence may have an intermediary effect in the model, so we added the interaction terms of “actual value of income” and “NDVI” and “actual value of income” and “park distance” to the model. The reason why the interaction term of “actual value of income” and “VGI” is not included is that people usually do not choose their residence according to street greening, and the street greening is rarely affected by the change of built environment. This may also be the reason why the coefficient symbol of the variable “place” in the third model has not changed.

It is seen that “place” may influence the health effect of green space. Since this variable reflects the individual income and the green space status of a community, which also relates to income, Model 4 included green space-related variables and the interaction of income and green space. Given the lack of correlation between the VGI and income and the insignificant coefficient of “park area,” only “income × distance” and “income × NDVI” were included. In Model 4, the coefficient of “distance” was negative and significant, while the coefficient of “income × distance” appears to be positive and significant. This shows that income affects the effect of distance from the park on the BMI. It is said that in the low-income group, the farther the distance to the park, the greater the probability that the BMI of the residents is normal, while in the high-income group, the closer the distance to the park, the lower the probability of obesity of the residents. This may be because, among the high-income groups, people are more willing to go to the nearest park for exercise activities. Increasing income may discourage the “distance” variable from obesity restraints. Calculating the income threshold, a longer distance promoted the deterioration of obesity when individuals' income exceeded an actual value, and that is an income level that all employed individuals could achieve. Thus, for employed residents, shortening the distance to parks helped them stay healthy, while for those who were unemployed, the opposite was true. The coefficient of “NDVI” was positive and significant, while the coefficient of “income × NDVI” was insignificant. This shows that income also affects the effect of vegetation coverage on the BMI, and in the lower-income group, a high NDVI increases obesity, while in the higher-income group, a high NDVI environment makes people's BMI more normal, while the effect of the moderation of income could not be evaluated. “VGI” yielded negative and significant coefficients, just like in Model 3. Residents with higher VGI were more unlikely to be overweight or obese.

## Conclusion and Discussion

Differentiated outcomes of regression in BMI groups may result from the uneven distribution of individual income and green space of communities. In this study, the VGI was introduced as the index of green space to study its influence on community residents' BMI, and the effect of green space on residents' health was evaluated from a new perspective. Through regression analysis, this study reveals the relationship between the VGI and residents' health. This study argues that street scenery has an impact on residents' health. The greening in street scenery reflects the overall level of street greening around the community, and street greenery is an important aspect of green space that residents come into contact with daily. The VGI is more closely correlated with residents' health than park green space or the NDVI. By comparing with the NDVI data widely used in the previous literature and the distance from the community to the park, this study reveals that the VGI yields different outcomes from the NDVI and green space distance, i.e., vertical greening has an impact on the health of residents. Because VGI, NDVI, and urban park spaces affect and measure the characteristics of green space from different approaches, the VGI is a good supplement to the above in the study of the health effects of existing green space. We should comprehensively understand the effect of green space on health from the aspects of horizontal and vertical greenery as well as the accessibility of green spaces.

We focused on the health represented by BMI and drew the above conclusions. However, because of the complexity of health evaluation criteria, there are defects in using a single BMI index to evaluate the health status. More health indicators should be used to further strengthen the conclusion of this study. According to the sample collection process, because of the high proportion of the elderly population in the research community, the aging of the community was more significant. Before the regression analysis, the community already somewhat contained aging characteristics. It is known that the impact of community green space, including VGI and NDVI, on residents' health may improve residents' health level by promoting activities in green space. To study the effect of activity time on the health of green space, we can introduce the variables of residents' activity time and their intersection with the characteristics of green space in the future.

The initial objective of this research was to examine the association of neighborhood greenness and residents' obesity in a high population density context. There are three different types of greenness features involved in our study, including green access, green exposure, and the VGI. Our research was built upon a large-scale survey from the WHO, and some key findings from a multilevel mixed-effects ordered logistic regression estimation are reported as follows. (1) It was found that, of the three types of greening levels, only the VGI consistently posed a negative effect on overweightness and obesity, indicating that eye-level neighborhood greenness could efficiently promote the physical activity of residents aimed to control their weight. (2) There was no significant relationship between the NDVI and the BMI in the beginning, but after we added the intersection of income and the NDVI, we found that the high-income group with better vegetation in their communities might have a better health status. (3) It was found that green proximity had a significant effect on overweightness and obesity, but the negative correlation was unexpected, indicating that people closer to the park were associated with a higher likelihood of obesity. When we accounted for the intersection of income and distance, the above issue was well-explained. The results showed that Shanghai residents with high income and normal body weights live close to city parks. Our results meaningfully complement the existing literature in two respects.

First, we provided evidence that neighborhood greenness has a preventive effect in weight management for high-density areas. It was found that all aspects of horizontal and vertical green levels and proximity to the same have an impact on body weight in case of a lack of green space resources per capita. Although we found that the correlations of some variables were incorrect, it is because China was established in the early stage of rapid urbanization, and a mixed U-shaped curve was formed in which the rich are overweight and the poor are becoming obese (45, 46).

Second, our results also confirmed that the VGI of the deep learning approach using Baidu Street View images could effectively capture the eye-level greening features in high-density areas of the population (21); such VGI can effectively promote walking and other physical activities to prevent obesity. The traditional measurement method is mainly based on the two-dimensional space, assuming that residents will use the urban green space nearby, but this may not be true for individuals using green space for recreation (47). Such VGI based on big data provides the possibility of measuring the greening level in 3D, and street view and satellite-derived green space measures represent different aspects of natural environments (48). Many studies have shown that this type of perceived greening can improve physical activities (49).

Given this information, we acknowledge that there are still some limits in this study. For instance, the sage survey fails to perfectly represent the overall greening condition of Shanghai. Moreover, the NDVI feature may vary across seasons, and the VGI measures the green space along the road network based on street view images. We acknowledge that such data are affected by the sampling distance and the street view data themselves. Also, the street view image is formed across the year, and it is hard to identify the season of images, which makes the time of VGI data uncertain.

Finally, our study offers important implications for future policies. China's green space planning system should be further improved, considering the supply and management of vertical greening. In such a high-density built environment area as Shanghai, the establishment of a new green space is a huge challenge for planners. To improve vertical greening, we can try to increase the used vertical space, such as building elevation as the target zone. Road greening can be developed to improve road users' feelings in places where plants near residential areas will not be intrusive to urban traffic efficiency. Although large park green space has a strong externality and can increase housing prices (50), our research found that the size of the park has nothing to do with the weight of residents. Therefore, health-oriented planning should promote the construction of a green space system and increase the type of green space and encourage a small and decentralized greenway network system, which is conducive to multiple physical activities, such as walking, cycling, and others. Meanwhile, it is necessary to strengthen the community vegetation coverage of living environments.

## Data Availability Statement

The raw data supporting the conclusions of this article will be made available by the authors, without undue reservation.

## Author Contributions

YX: writing - original draft, writing – review, and editing. YZ: formal analysis. YS: methodology for deep learning. PT: data analysis. XK: funding acquisition, validation, conceptualization, and supervision. All authors contributed to the article and approved the submitted version.

## Conflict of Interest

The authors declare that the research was conducted in the absence of any commercial or financial relationships that could be construed as a potential conflict of interest.
